# A Much-needed Organization

**Published:** 1897-06

**Authors:** 


### A MUCH-NEEDED ORGANIZATION.

Many creditable representatives of the veterinary profession of St. Louis, Mo., have organized a local association which we are sure, from the selection of its officers, will be a very beneficial one in matters of interest to the profession, and will be the cause of enlightening public sentiment on such matters. The Journal extends to the organization its best wishes for a successful career.

---

Governor Black, of New York State, has signed the Administration Bill of the Empire State, in which occurs the following important announcement: "No tuition fee shall be required of students pursuing the regular veterinary course who, at the time of admission to the State Veterinary College, are legal residents of this State." This offer to prospective students, residents of New York State, will make it a very grave matter for other colleges which are almost wholly dependent for support on the fees they receive, as in many instances, in both the New York City schools, which receive a large number of students from both city and State. We take it this concession has been made inasmuch as a fixed appropriation referred to in a former number of the Journal has been placed annually at the disposal of the New York State Veterinary College for the yearly expenses.

---

Inspecting veterinarians of the Boston Horse Show are coming in for a good deal of scoring on account of their holding up a number of the horses in the ring for suspected unsoundnesses.

---

We announce on another page of this issue the publication of several important works by the well-known publishing house of William R. Jenkins. The books announced are very important ones, and will no doubt be gratefully received by the veterinary profession, especially by the faculties of our colleges.

Bremen, Germany, has issued orders that all horses entering that port must be submitted to a veterinary examination, and the fee for this service must be paid by the consignee. This system will, no doubt, lead to a closer inspection of these animals before shipment from American ports.

